# Can adopting lean startup strategy promote the sustainable development of new ventures? The mediating role of organizational iterative learning

**DOI:** 10.1371/journal.pone.0290849

**Published:** 2023-08-30

**Authors:** Kai Zhuge, Huitao He, Yongzhi Yuan, Peiting Sun

**Affiliations:** 1 Business School, Soochow University, Suzhou, Jiangsu, China; 2 School of Political and Public Administration, Soochow University, Suzhou, Jiangsu, China; Sichuan Agricultural University, CHINA

## Abstract

When high uncertainty becomes the norm in entrepreneurship, entrepreneurial failure often becomes the first natural barrier that new ventures face. In dynamic environment, there is still a lack of clear answers on what strategic orientation new ventures need to adopt to improve organizational learning efficiency and achieve sustainable development. Lean startup theory believes that the entrepreneurial process is also a process where organizational cognition is constantly iterated and updated, which drives entrepreneurs to explore business opportunities through iteration learning and early customer insight. The paper aims to describe and examine the relationship between lean startup strategy, organizational iterative learning, and sustainable development of new ventures. This model is tested on the survey data of R&D departments of 325 technology new ventures in China. The research results show that: lean startup strategy can positively affect sustainable performance of new ventures; organizational iterative learning plays a mediating role in the relationship between lean startup strategy and sustainable development; market dynamics positively moderate the relationship between organizational iterative learning and sustainable development, while technology dynamics negatively moderate this relationship; furthermore, the two also moderate the process of lean startup strategy influencing sustainable development through organizational iterative learning, and that moderated mediating effect is significant. The research results reveal that entrepreneurs should deepen lean startup practices in new business layout, advocate the iteration and output of advanced knowledge, help enterprises establish proprietary knowledge barriers, and achieve sustainable development.

## 1. Introduction

Under the dual background of the new industrial revolution and the global pneumonia epidemic, transformation has become an important means for enterprises to enhance new momentum and seek profitable growth. The deep integration of the digital economy represented by new technologies such as cloud computing and artificial intelligence and the traditional economy has not only promoted the overall transformation of enterprises, but also intensified the complexity of the entrepreneurial environment. The instability of the environment reduces the effectiveness of the "self-centered" and "genius imagination" patterns advocated by traditional entrepreneurial thinking [1, 2]. Meanwhile, the lack of entrepreneurial learning experience makes entrepreneurs easily fall into the "trap" of overconfidence and blind entrepreneurship [3, 4], which leads to irrational decision-making, even entrepreneurial failure. How to correct the misunderstanding of entrepreneurs’ strategic thinking and guide new ventures to establish entrepreneurial benchmarks guided by value needs in the "fog" has become an important issue related to the sustainable development of new ventures. [5] proposed that lean startup (LS) is an entrepreneurship theory based on market orientation, which optimizes and improves initial products through organizational dynamic learning and valuable market feedback, so as to obtain new business cognition. Studies have shown that the lean startup strategy (LSS) can effectively improve the product development efficiency [6], bring new ideas and creativity to enterprise intrapreneurship [7, 8], and then create new market opportunities [9–11].

In fact, due to the lack of initial resources and the internal learning mechanism that has not yet been established, the premature death of new ventures always occurs on the logical issue of "what do customers really want" [12, 13]. This is not only because managers explore what key knowledge companies need to learn through the establishment of a demand-oriented initiative order [14], but also because they build crisis awareness into the sustainability of entrepreneurship [15], because the lack of key knowledge can easily lead to products not being accepted by the mainstream market during the promotion stage [16], making the enterprise lose its sustainable resilience in the early stage. As the "user-centered" pain point logic is increasingly favored by entrepreneurs, when initial resources are at a disadvantage, enterprises can establish a first-mover advantage through responding to customer needs immediately, learn how to optimize products or services in the process of interacting with customers, and gradually gain market share through repeated trial-and-error learning. This behavioral process of focusing on market experience and emphasizing organizational active learning is called "organizational iterative learning" (OIL). Organizational learning theory believes that organizations can create, exchange and iterate knowledge through the active learning process, stimulate the organization’s product iteration capability and innovation efficiency [17]. [18] pointed out that interactive learning with customers is conducive to the rapid integration and utilization of fragmented resources by new ventures to obtain unexpected benefits and achieve innovation catch-up. Organizational iterative learning emphasizes mining potential customer needs through responsive learning, realizing innovation and optimization in interactive learning, improving customer value through trial-and-error learning, and finally achieving market penetration and occupation in continuous learning, which can well make up for the neglect of user needs analysis from a strategic-oriented perspective, so that enterprises no longer stick to assumptions about demand. Instead, make decisions in communication and feedback with customers and transform original ideas into products with commercial value through multiple iterations [19], breaking away from the vicious circle of "the harder you work, the more you fail". As an important precursor and strategic background of the iterative learning process, lean startup can fully coordinate and manage the resource relationship of new ventures in the early stage of entrepreneurial learning and stimulate value creation through the reorganization of various capabilities.

In recent years, the relationship between lean startup strategy and new ventures has received extensive academic attention [20]. Existing studies have discussed it from multiple perspectives such as lean practice [21], intrapreneurship [8, 19], and business models [22], emphasizing that lean startup strategy can well alleviate the resource scarcity dilemma of new ventures. Considering that lean startup is a prerequisite for organizations to carry out iterative R&D and learning behaviors, as well as a reference point that influences organizations to make behavioral decisions, it is necessary to understand the impact of lean startup strategies on organizational iterative learning. Based on this, we believe that the existing research still has the following deficiencies:

(1) The lean thinking of new ventures is reflected in the bottom-up iterative process. Entrepreneurs need to deepen the customer base through iterative learning and use feedback to enhance the ability to learn in reflection. However, few studies have explored the role of lean startup strategy on organizational sustainable development from the perspective of iterative learning process.
(2) In the process of new ventures promoting iteration through user feedback, environmental dynamics is an important contingency factor, which is a perception of the evolution of stakeholder’s consciousness and technological updates between generations [23, 24]. Entrepreneurs’ grasp of environmental value is an important component of effectively predicting consumer preferences and making decisions. However, existing literature focuses more on the static or closed level when considering environmental factors, and does not pay enough attention to its dynamics.
(3) The impact of lean startup is complex and uncertain, and it may be premature for academic consensus that it is beneficial for entrepreneurial performance [6]. This possibility suggests that the impact of lean startup strategies on other variables, such as organizational iterative learning, should be explored to further demonstrate how lean enterprises affect entrepreneurial behavior through iterative learning.

In sum, there are still some theoretical gaps in explaining how lean startup strategies can build a bridge to entrepreneurial sustainability under the influence of organizational iterative learning and environmental dynamics. Therefore, this paper aims to explore whether lean startup strategy can affect the sustainable development of new ventures, constructing a theoretical model with organizational iterative learning as the mediating variable and environmental dynamics as the moderating variable, and analyze the mechanism and boundary conditions of iterative learning on the sustainable development of new ventures, which has important theoretical significance for further enriching the mechanism between lean startup strategy and new ventures.

Therefore, our team designed this study to determine whether lean startup strategies affect the sustainable development of new ventures. The empirical data was gathered using multi-source and multi-wave questionnaire survey from 325 Chinese technology new ventures and analyzed using least squares regression. The main conclusions were as follows: Lean startup strategy can positively affect the sustainable development of new ventures; organizational iterative learning plays a mediation role in the relationship between lean startup strategy and sustainable development of new ventures; market dynamics positively moderates the relationship between organizational iterative learning and sustainable development, while technology dynamics negatively moderates this relationship; and the two also moderate the process of lean startup strategy influencing sustainable development through organizational iterative learning, that is, the moderated mediation effect is significant.

This article supplements and expands the research on the impact of lean startup strategy on the sustainable development of new ventures from the following aspects. Firstly, based on lean startup theory, we proposed the theoretical model of "lean startup strategy-organizational iterative learning-sustainable development of new ventures" from the perspectives of response learning, interactive learning, trial-and-error learning, and continuous learning, explaining the iterative learning process and its driving effect on new ventures. Secondly, we have further enriched the decisive factor research of the sustainable development of new enterprises, and provide a new interpretation for new venture growth from the perspective of lean startup strategy. Finally, we incorporated the interactive impact of environmental dynamics on the sustainable development of new ventures. Under the disturbance of the external environment, organizational iterative learning promotes continuous improvement and adjustment of products, enabling entrepreneurs to gradually clarify product value and sustainable entrepreneurial direction during iteration.

The rest of our paper are organized as follows. In section 2, we provide a literature review and theoretical assumptions. In section 3, we discuss sample sources and variable measurements. In section 4, we present the results of the reliability and validity test, the common method bias test, and the hypothesis test. Finally, in section 5, we present the conclusions, theoretical contributions, managerial implications and future prospects.

## 2. Literature review and hypotheses development

### 2.1 Lean startup theory (LST) and organizational iterative learning (OIL)

The highly uncertain entrepreneurial environment has exacerbated the complexity of the relationship between supply and demand in the market, and the differentiation of entrepreneurial opportunities and risks has become more prominent. This puts forward higher requirements for the decision-making quality of entrepreneurs, making them hope to reduce the trial-and-error cost and the entrepreneurial risks through effective means [25]. Based on the theoretical practice and philosophy of "Lean Production" (LP), [5] proposed the lean startup theory, which suggests that new ventures should follow such an entrepreneurial procedure in high uncertainty environments, by providing customers with a minimum viable product (MVP), continuously optimizing products through active trial and error experiments and hypothesis verification, and creating customer value and eliminating waste in the entrepreneurial process as quickly as possible.

The core idea of lean startup theory can be summarized as constructing a confirmatory learning cycle of "development-measurement-learning" [5, 26], driving organizational cognitive updating and knowledge iteration. The entrepreneurial process of a lean enterprise is also a process of continuous learning for the organization [27–29], and the construction of its iterative capability needs to be based on organizational learning [30]. Lean startup takes " confirmatory learning" and "simple model—interaction and trial and error—adjustment and optimization" as customer development paths, which is the organizational iterative learning process of "response—interaction—trial and error—optimization". Firstly, lean startup theory encourages enterprises to quickly respond to customer needs in the early stage of entrepreneurship [31, 32], and develop the simplest product prototype with minimal cost; Secondly, push the product prototype to the target market to achieve product-customer interaction, and continuously verify whether the product meets the customer’s needs in the early stage [5, 33]; Thirdly, strengthen the trial-and-error learning ability through frequent customer contact and receiving feedback in the middle and late stages, and perceive changes in customer needs faster than competitors [26, 32, 34]; Finally, in the dynamic and continuous learning process, provide higher quality products in a shorter time to achieve a high "service-market" fit and maximize customer value.

Lean startup theory tries to help entrepreneurs break through the constraints of traditional thinking and the cocoon of "information asymmetry", promoting entrepreneurial organizations to establish an iterative learning order in uncertain entrepreneurial situations. Organizational iterative learning enables lean enterprises to use platform interaction, product feedback, user tracking and other learning paths to achieve cognitive iteration, that is, to understand more precisely what customers want; and then through agile R&D, small steps, trial and error accumulation and dynamic optimization and other learning processes to achieve product or service iteration, that is, to launch more precisely products that customers want, in the iterative cycle to promote sustainable development of entrepreneurship.

### 2.2 Lean startup strategy and sustainable development of new ventures

[11] believe that the goal of lean startup lies in improving development method of enterprises. The guidance of user logic enables entrepreneurs to strengthen the control over the staged goals of new ventures, use the least cycle and investment to launch minimum viable product, avoid unnecessary waste in the product and business development processes, and stop enterprise activities that do not create value. At each product launch, the impact of the product on the customer base and the customer’s suggestions for the product are immediately evaluated, based on the evaluation results, the correctness of the basic assumptions is judged [20], and the goals of the next product development work are determined. This can greatly narrow the gap between requirements and cognition, thereby avoiding the waste of resources caused by cognition differences and reducing entrepreneurial risk [35]. Specifically, the lean startup strategy further formalizes and visualizes the product development process [33], entrepreneurs can grasp the lean level of the enterprise by monitoring performance indicators such as resources, costs, and green, so that the enterprise can increase the chance of entrepreneurial success with less marketing expenses.

Different from the traditional entrepreneurial model that transforms self-imagination into reality, the lean startup strategy provides new ideas for new ventures to ease resource constraints and capture entrepreneurial opportunities. On the one hand, the lean startup strategy can guide entrepreneurs to effectively identify the high-quality resources needed for entrepreneurship, and make timely resources patchwork to ensure the availability and liquidity of limited resources [30, 36]; On the other hand, the lean startup strategy’s dedication to feedback helps to increase customers’ willingness to share information, and the improvement in the quality of interaction between enterprises and customers also makes it easier to track pain points in product design. Therefore, establishing a deep relationship with customers can help new ventures obtain reliable feedback data at a low cost, enabling entrepreneurs to better understand the market and future changes, compensating for the initial disadvantages of latecomers by matching customer value needs with entrepreneurial capabilities [37, 38].

It can be seen from the above that lean startup strategy no longer pursues technological innovation and perfection, but focuses more on the establishment of market orientation [26], its core is to create and enhance customer value, and to achieve sustainable development through lean circulation. The search, acquisition, and sharing of demand information are key to achieving catch-up in lean enterprises [39], new ventures should actively create an interactive business circle with stakeholders, deepen customers’ sense of participation and emotional investment in product or service improvement through lean startup strategy, enhance brand cohesion, and achieve sustainable development. The following hypothesis is based on the preceding discussion:

*H1*: Lean startup strategy positively affects sustainable development of new ventures.

### 2.3 The mediating effect of organizational iterative learning

Implementing lean practice in enterprises is a complex process [40]. Lean entrepreneurship is not just about optimizing individual procedures, but seeking for the optimization of the sustainability of the entire enterprise from the perspective of change [41]. In the early stage of entrepreneurship, the strategy of lean startup is to encourage enterprises to accept failures, learn from experience through failures, promote knowledge iteration, and timely discover and abandon technical routes that do not meet market expectations. However, given the explicit differences in effectiveness between learning modes, organizations are inevitably unable to maintain balanced learning attention for a long time and efficiently, and the issue of ‘the impact of the balance of learning behaviors at different stages on organizational performance’ has yet to be revealed [42]. At the same time, due to intensified uncertainty of the market environment, lean startup emphasizes tightening resources, which leads entrepreneurs to be more cautious in resource investment; In addition, the R&D process of new products is becoming more and more complicated and extremely vulnerable to technological changes, which also contributed to the emergence of organizational iterative learning mechanisms in the context of lean startup.

The value creation of organizational iterative learning lies in transforming discrete demand information in the market into learning signals through active learning, and the new knowledge learned is used to iterate outdated knowledge within the organization. It advocates agile awareness, requires enterprises to respond and interact with changes in consumer needs in a timely manner [43], thereby solving the "unknown needs" gap in entrepreneurship; organizational iterative learning also emphasizes customer engagement and reflective learning, which means having the ability to explore and innovate new knowledge that customers need [44, 45]. The advantages of this learning mode are reflected in the small burden on the enterprise as a whole, ease of grasping psychological needs of customers step by step, improving product stickiness, and provide a high-efficiency path for low-cost R&D of enterprises. The lean startup strategy encourages entrepreneurs to actively propose and validate pain point hypotheses, and the premise of validation lies in iterative learning of feedback information, on the one hand, organizational iterative learning can enable enterprises to clarify the needs or tendencies implied in feedback by efficiently mining and transforming external knowledge resources, thereby quickly narrowing the gap between actual needs and solutions; On the other hand, in the middle and late stages of entrepreneurship, enterprises can establish a fitting model based on previous iteration data, determine the short-term demand trend to lock in the future output value of products, thereby enhancing the ability of enterprises to resist uncertain risks and driving sustainable development.

For example, Xiaomi has realized the original accumulation of user groups through extensive penetration of forum enthusiasts. The analysis of a large amount of feedback information has enabled Xiaomi to improve performance of smartphones in a short term, at the same time increase the market share of MIUI system and establish a sustainable ecology. As [46] pointed out, the interaction between entrepreneurs and users stimulates the development and iteration process of resources, so that users can clearly feel the process of increasing value. Therefore, organizational iterative learning is not only an important learning mode product development, but also an important means to cope with market competition and environmental mutations. Organizational iterative learning enables entrepreneurs to focus on the assessment and prediction of customer needs, relying on market forces to solve problems that enterprises cannot solve [47]. The lean startup strategy enables enterprises to further clarify market trends, correct errors in the R&D process, provide guarantee and learning foundation for organizational iterative learning of "response-interaction-trial and error-optimization", continuously stimulate new ideas, reduce entrepreneurial uncertainty, and promote sustainable development of entrepreneurship. The following hypothesis is based on the preceding discussion:

*H2*: Organizational iterative learning can play a mediating role between lean startup strategy and sustainable development of new ventures.

### 2.4 The moderating effect of environmental dynamics

Environmental dynamics refers to the unpredictability of changes in the external environment of the enterprise, including changes in the market environment and technological environment over time. At present, with the breakthrough innovation of digital technology and the individualization of user needs, the entrepreneurial environment is becoming increasingly complex and unpredictable. Scholars have confirmed that the performance of new ventures varies with the degree of environmental disturbance [48]. Due to the existence of organizational routines, mature enterprises tend to utilize their strong knowledge base to maintain competitiveness when facing dynamic environments. But for new ventures, high environmental turbulence can easily affect their weak knowledge structure [49], thereby affecting sustainable development.

From the perspective of market dynamics, high market dynamics increase the difficulty for new enterprises to identify, search for, and obtain the necessary resources. Organizational iterative learning is an important way to deal with high market dynamics. It shortens the product development cycle of new ventures, and the low learning cost reduces the risk of market development, which undoubtedly improves the enterprise’s willingness to actively iterate to seize those innovations with high-return value, and ensures the sustainability of entrepreneurial activities [50]. At the same time, high market dynamics increase the demand for new ideas and new methods, and also increase the difficulty of knowledge creation. It is difficult for new ventures to cope with changes in the market environment relying on existing static knowledge. If they can timely carry out organizational iterative learning, master new information and technologies, and form a knowledge system that adapts to the environment, then the role of organizational iterative learning in promoting the sustainable development of new ventures will be more obvious.

From the perspective of technology dynamics, external technology updates are relatively rapid, technology lifecycle are shortened and easily eliminated. In this case, the technological innovation paradigm of enterprises is threatened by the external environment. Although enterprises can respond to technological iterations and establish a new knowledge base by investing more resources and costs, this will greatly increase entrepreneurial risks, especially for new enterprises, which cannot maintain high investment in technology R&D for a long time [51]. However, if the new enterprises chooses to rely on existing technologies to improve the status quo, instead of taking measures to break through the existing knowledge base, it will be difficult to develop new products or tap new customer needs, resulting in difficulty for the enterprise’s innovative achievements to be recognized by the market, and thus reducing sustainable performance. The following hypothesis is based on the preceding discussion:

*H3a*: Market dynamics can positively moderate the relationship between organizational iterative learning and sustainable development of new ventures.
*H3b*: Technology dynamics can negatively moderate the relationship between organizational iterative learning and sustainable development of new ventures.

### 2.5 The moderated mediation

Combining H3 and H4, our study further proposes a moderated mediation model. Organizational iterative learning plays a mediating role in the relationship between lean startup strategy and sustainable development of new ventures, but the size of its role depends on the level of environmental dynamics. When market dynamics is high, willingness of new ventures to iterate old knowledge and learn new knowledge increases, and lean startup strategy effectively reduce the cost and risk of enterprises’ search and integration of market knowledge, improve the efficiency of organizational iterative learning, and increase the advantage of sustainable development. When technology dynamics is high, Regardless of whether new ventures spend their main energy on coping with technology iteration or maintaining the technology status quo to improve customer feedback, it will lead to uneven resource allocation and limited opportunity identification, thus hindering the role of organizational iterative learning. Even if lean startup strategy drives the organizational iterative learning process, high technical dynamics will make the effect of iterative learning less prominent, resulting in a negative impact on sustainable development. The following hypothesis is based on the preceding discussion:

*H4a*: Market dynamics can positively moderate the process of lean startup strategy influencing sustainable development of new ventures through organizational iterative learning.
*H4b*: Technology dynamics can negatively moderate the process of lean startup strategy influencing sustainable development of new ventures through organizational iterative learning.

**Fig 1:**
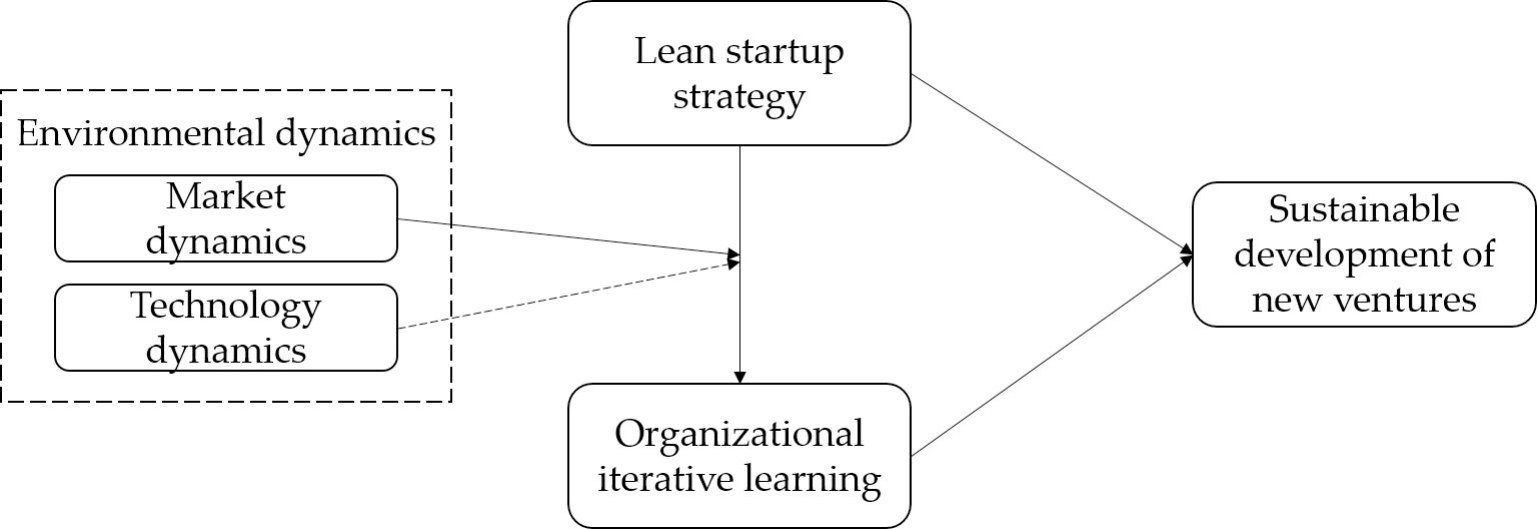
Theoretical model. Notes: The solid line represents positive relationship and the dotted line represents negative relationship.

Integrating the above relationships and all the hypotheses, H1–H4, the theoretical framework of this research is shown in Fig 1. Based on lean startup theory, we believe that new ventures require abundant market resources to achieve sustainable development and that lean startup strategy provides a reliable channel to obtain these resources, which significantly increases the efficiency of organizational iterative learning, enabling enterprises to develop and share key knowledge dispersed in stakeholder networks in the process of iterative learning and under dynamic environment, thereby stimulating new ventures to establish sustainable development advantages. Therefore, we propose assumptions that lean startup strategy can promote sustainable development of new ventures, that organizational iterative learning plays a mediating role in the impact mechanism of lean startup strategy on sustainable development, and that environmental dynamics can moderate the relationship between organizational iterative learning and sustainable development. Furthermore, we believe that environmental dynamics can moderate the mediating role of organizational iterative learning.

## 3. Methodology

### 3.1 Data sources

The context of this study is technology new ventures in China. While numerous studies on lean startup have been conducted in Western countries, only a few studies have focused on organizational learning issues on emerging economies, especially, those in China. As one of the highly potential emerging economies, China has been considered to play an important producer role in the past 20 years, with vibrant private economy and strong innovation atmosphere, and is the headquarters of many multinational companies. Under the increasingly sound policy system incentives, the demand for talent entrepreneurship continues to increase. Therefore, it is highly representative and feasible to choose Chinese new ventures as the research object.

To avoid sample selection bias and endogenous problems, we collected data in stages through combining online and offline methods. Before the formal survey, the questionnaire was further improved through feasibility evaluation by experts in the field of lean startup and also a pre-survey of 15 MBA students to ensure the content validity of the questionnaire. In order to ensure the participants’ overall understanding of the survey content, we investigated R&D department employees engaged in R&D, idea design, and operation, because these employees are directly related to the actual needs and requirements of customers, and undertake the tasks of creating new products, developing updated technologies and providing personalized solutions. Based on [52], this study selected technology new ventures that were more than one year old but less than eight years old as the survey targets. The sources of formal survey data are as follows: in 2022, we contacted the administrative committee of science and technology entrepreneurship parks in Yangtze River Delta cities to provide us with lists of enterprises and selected 380 enterprises with different sizes and industries by using random sampling method. Firstly, we entrusted the administrative committee to contact the human resources managers of these enterprises via e-mail and explain the research purpose and content. Subsequently, we entrusted the human resources manager to issue electronic or paper questionnaires to a colleague in the R&D department. Participants were required to fill in the last four digits of mobile phone number, which were used for questionnaire matching in the two-stage survey. In addition, we explained to the participants in the questionnaire that this survey is completely voluntary, the data is strictly confidential, and the data will only be used for academic research.

The questionnaire distribution process was divided into two stages. Lean startup strategy, organizational iterative learning and demographic information were measured in the first stage, and received 371 responses. Two weeks later, the scales of market dynamics, technology dynamics and sustainable development were sent to those employees who participated in the first stage survey, and received 356 responses. We matched the two-stage questionnaires and eliminated samples that did not meet the requirements for reasons such as being incomplete and having been filled out in an irregular way. A total of 325 usable questionnaires remained, for an effective response rate of 91.30%. The distribution characteristics of the sample enterprises are shown in Table 1.

**Table 1:** Distribution of sample enterprises.

Features	Categories	Number	Percentage (%)
Enterprise age	1~3	102	31.38
	3~5	147	45.23
	5~8	76	23.38
Enterprise nature	State-owned	34	10.46
	Private-owned	257	79.08
	Foreign-owned	26	8.00
	Other	8	2.46
Staff size	≤ 20	43	13.23
	21~50	79	24.31
	51~100	127	39.08
	101~200	51	15.69
	>200	25	7.69
Industry involved	Biomedicine	37	11.38
	Information software	62	19.08
	New energy	45	13.85
	New material	32	9.85
	High-end equipment manufacturing	78	24.00
	Energy and environment	23	7.08
	Others	48	14.77

### 3.2 Survey instruments

All the scales used in this research were selected from mature Western literature and had exhibited good reliability and validity. The scales were first translated into Chinese and then translated back into English, and some items were appropriately modified and adjusted according to the agility characteristics of new ventures and the actual research situation. A management professor with overseas work experience was consulted by us to ensure that the scale can be accurately translated into Chinese. A Likert 5-point scale was adopted for all scales in this study, in which ‘1’ means strongly disagree and ‘5’ means strongly agree.

Lean startup strategy: the measurement of this variable mainly referred to the research of [9], which included 13 items. Such as ‘We encouraged our current clients to refer our products to new clients to find out what can be done to improve the process’, ‘We followed guerilla tactics in marketing’, etc.

Organizational iterative learning: the measurement of this variable mainly referred to the research of [53–55], which is measured from four aspects: responsive learning, interactive learning, and trial-and-error learning and continuous learning. The scale includes 11 items, such as ‘we often conducted responsive design based on feedback’, ‘we encouraged users to feedback their opinions on products or services to the enterprise’, etc.

Market dynamics and technology dynamics: the measurement of this variable is mainly based on the research of [56], which included 3 items for market dynamics and 3 items for technology dynamics. Such as ‘Users tend to look for new products or services’, ‘It is difficult to predict the future technological development trend of the industry’, etc.

Sustainable development: sustainability depended on three fundamental pillars: economic, environmental and social, many studies have supported this view. Therefore, the measurement of this variable is mainly based on the research of [57], which included 3 items. Such as ‘we provide technology, management, and financial assistance to solve social problems’, ‘We have implemented a quality and environmental management system such as ISO18000/14000’, etc.

Control variables: enterprises of different age, nature and size, and different industries may show different characteristics, which in turn may influence sustained competitive advantage [58]. Therefore, these variables were included as control variables.

### 3.3 Analytical technique

In this study, SPSS 20.0 software was used to verify the reliability and validity of the questionnaires and test the model’s correctness through the hierarchical regression method under the condition of including control variables. Second, in order to further observe the relationship between variables, we used Mplus 8.0 software through the three-step method to test the mediation mechanism of the model and used the bootstrap method to test the moderated mediation effect. Third, in order to ensure the robustness of the model and better understand the impact of lean startup strategy and organizational iterative learning on new venture growth, we used PROCESS 3.3 to calculate confidence intervals for the mediation effect to determine whether the relevant assumptions were valid.

## 4. Results

### 4.1 Common method bias test and confirmatory factor analysis

To control for the influence of common method bias on the result, one of the procedural measure adopted in this study was that the order of variables was randomly adjusted, and the other was that the confidentiality and anonymity of the questionnaire were guaranteed to the subjects, thus reducing the systematic error caused by artificial covariation. Subsequently, this study used CFA and ULMC methods to detect common method problems. In confirmatory factor analysis (CFA), if the fit indices of the single-factor model is the worst compared with other multi-factor models [59], it proves that common method bias is not serious. Table 2 shows that the fitness of the Model 5 was the worst. Furthermore, [60] proposed to check common method bias by controlling for the effects of an unmeasured latent method factor (ULMC). That is, a common method factor is added to baseline model so that it has the same loading value on all items. The fit indices of the Model 1 + method factor was χ2/df = 2.277, CFI = 0.941, TLI = 0.912, RMSEA = 0.076, SRMR = 0.069, compared with the Model 1, the amplitude of change was within an acceptable range, indicating that the common method bias was not serious.

**Table 2:** Confirmatory factor analysis.

Model type	χ^2^	df	χ^2^/df	CFI	TLI	RMSEA	SRMR
M1: LSS, OIL, MD, TD, SD	703.420	309	2.276	0.941	0.913	0.074	0.066
M2: LSS, OIL, MD + TD, SD	844.703	314	2.690	0.826	0.805	0.120	0.074
M3: LSS + OIL, MD + TD, SD	990.872	321	3.087	0.780	0.760	0.133	0.076
M4: LSS + OIL, MD + TD + SD	1058.905	323	3.278	0.758	0.738	0.139	0.077
M5: LSS + OIL + MD + TD + SD	1493.928	324	4.611	0.616	0.584	0.175	0.132

Notes: N = 325, LSS = lean startup strategy, OIL = organizational iterative learning, MD = market dynamics, TD = technology dynamics, SD = sustainable development, the same below.

### 4.2 Reliability and validity analysis

First, the Cronbach’s α of each variable were more than 0.7, which is higher than the acceptable threshold value, indicating that the reliability of the scale was good. Second, according to Table 3, the minimum value of CR was 0.766 more than 0.7, and the minimum value of AVE was 0.594 more than 0.5, meaning both were higher than the threshold requirements, indicating that the aggregation validity of the model was acceptable. Finally, we tested the discriminant validity between the two variables, from Table 2, the fit indices of the Model 1 all met the fitness standard and were superior to other multi-factor models. And as shown Table 3, the square root of AVE of each variable was more than correlation coefficient among all the latent variables, indicating that latent variables have good discriminant validity.

**Table 3:** Descriptive statistics and correlation analysis.

Variables	CR	AVE	1	2	3	4	5
1. Lean startup strategy	0.807	0.620	0.787				
2. Organizational iterative learning	0.816	0.734	0.335**	0.857			
3. Market dynamics	0.837	0.643	0.208**	0.229**	0.802		
4. Technology dynamics	0.801	0.651	0.467**	0.383**	0.123*	0.807	
5. Sustainable development	0.766	0.594	0.306**	0.463**	0.447**	0.323**	0.771
Mean	/	/	3.580	3.981	3.920	4.017	3.540
SD	/	/	0.825	0.766	0.739	0.747	0.740

Notes

*means p<0.05

** means p<0.01; diagonal value are the square root of AVE.

### 4.3 Descriptive statistics and correlation analysis

As shown in Table 3, descriptive statistics and correlation analysis were measured for all variables, and there was a significant correlation between the focal variables. The correlation between lean startup strategy and sustainable development was positive (β = 0.335, p<0.05), and lean startup strategy was positively correlated with organizational iterative learning (β = 0.306, p<0.05); organizational iterative learning was positively associated with sustainable development (β = 0.463, p<0.05). Although these results preliminarily verified the hypotheses, hierarchical regression analysis was necessary to accurately understand whether these hypotheses were supported.

### 4.4 Hypothesis test

Before the hierarchical regression analysis, all the focal variables were standardized in this study to reduce the influence of multicollinearity [61]. Under different conditions of dependent variables, Model 1 and 3 were set as baseline models that only comprised control variables. Firstly, we examined the direct effect between lean startup strategy and sustainable development of new enterprises. Subsequently, we tested the mediating roles of organizational iterative learning with respect to the effect of lean startup strategy on sustainable development. Thirdly, we verified the moderating effects of environmental dynamics on the relationship between organizational iterative learning on sustainable development. Finally, the moderated mediation effects were examined.

Firstly, the results of the direct and indirect regression analyses were shown in Table 4, lean startup strategy had a positive effect on sustainable development of new enterprises (β = 0.294, p<0.001, Model 4), compared with Model 3, addition of the lean startup strategy improves the explanatory power of the model by 13.2%. Therefore, hypothesis 1 was supported, the direct effect was significant.

**Table 4:** Regression results for direct and indirect effects.

Variables	Organizational iterative learning		Sustainable development			
	Model 1	Model 2	Model 3	Model 4	Model 5	Model 6
**Control variables**						
Staff size	0.064	0.052	-0.023	-0.036	-0.035	-0.027
Enterprise age	-0.019	-0.013	-0.060	-0.057	-0.056	-0.052
Industry involved	-0.076	-0.071	0.113*	0.127*	0.124*	0.094
Resource constraints	0.083	0.087	0.175*	0.163*	0.170*	0.159*
**Independent variable**						
LSS		0.317***		0.294***		0.204**
**Mediator**						
OIL					0.447***	0.373***
R^2^	0.034	0.267	0.086	0.219	0.227	0.246
Adj_R^2^	0.022	0.256	0.075	0.207	0.215	0.232
F	2.085*	4.147***	1.932*	4.039***	4.364***	5.024***

Notes

* means p< 0.05

** means p< 0.01

*** means p< 0.001.

Secondly, according to Table 4, we can see that lean startup strategy positively predicted organizational iterative learning (β = 0.317, p<0.001, Model 2). When both lean startup strategy and organizational iterative learning were included in the regression equation, organizational iterative learning had a positive effect on sustainable development (β = 0.373, p<0.001, Model 6), while the effect of lean startup strategy on sustainable development decreased (β = 0.204, p<0.01, Model 6), thus suggesting that organizational iterative learning may partially mediate the relationship between lean startup strategy and sustainable development. Hypothesis 2 was thus supported, the mediating effect was significant.

**Fig 2:**
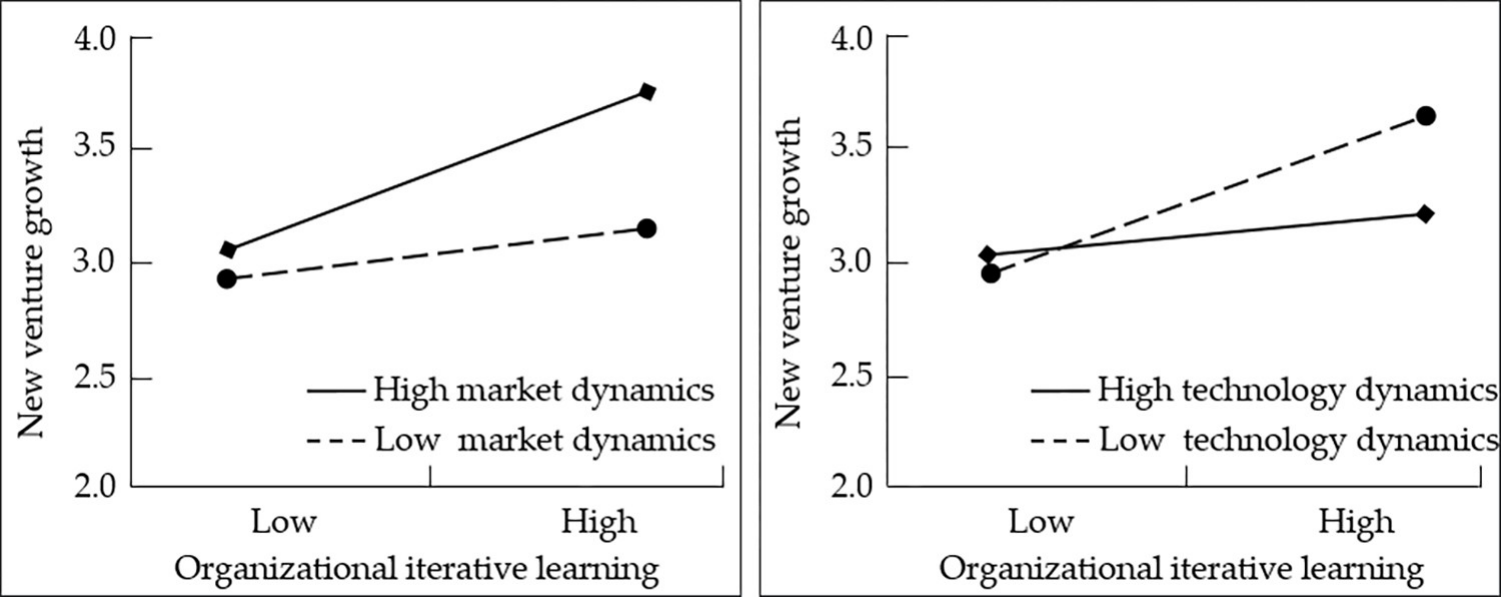
The moderating effect of environmental dynamics.

Thirdly, the regression analysis results concerning the moderating effect were shown in Table 5. The interaction term of organizational iterative learning and market dynamics had a positive effect on sustainable development (β = 0.192, p<0.01, Model 8). According to [61], we plotted the effect of organizational iterative learning on sustainable development under different levels of environmental dynamics, and the results are shown in Fig 2. The positive effect of organizational iterative learning on sustainable development was stronger when market dynamics was higher, Hypothesis 3a was thus supported.

**Table 5:** Regression results for moderating effects.

Variables	Sustainable development			
	Model 7	Model 8	Model 9	Model 10
**Control variables**				
Staff size	-0.031	-0.033	-0.029	-0.041
Enterprise age	-0.059	-0.048	-0.052	-0.044
Industry involved	0.128*	0.085	0.103	0.098
Resource constraints	0.175*	0.143*	0.161*	0.151*
**Mediator**				
OIL	0.354***	0.305***	0.362***	0.339***
**Moderator**				
MD	0.283***	0.278***		
TD			0.154*	0.163*
**Interaction**				
OIL×MD		0.192**		
OIL×TD				-0.201**
R^2^	0.236	0.278	0.251	0.282
Adj_R^2^	0.226	0.262	0.243	0.266
F	5.212***	6.381***	4.360***	6.021***

Notes

* means p< 0.05

** means p< 0.01

*** means p< 0.001.

In the same way, the interaction term of organizational iterative learning and technology dynamics had a negative effect on sustainable development (β = -0.201, p<0.01, Model 10). Fig 2 illustrates the relationship between organizational iterative learning and sustainable development under different levels of technology dynamics. When technology dynamics was higher, the positive effect of organizational iterative learning on sustainable development was weaker. Hypothesis 3b was thus supported, the moderating effect was significant.

Finally, this research further examined whether market dynamics moderated the mediating effect of organizational iterative learning on the relationship between lean startup strategy and sustainable development. The results of the moderated mediation effect are shown in Table 6. When market dynamics was higher, lean startup strategy had a stronger indirect effect on sustainable development via organizational iterative learning (estimate = 0.110, BootSE = 0.054, 95% confidence interval [0.040, 0.218]). When market dynamics was lower, the indirect effect between lean startup strategy and sustainable development via organizational iterative learning was significant but below high market dynamics (estimate = 0.066, BootSE = 0.031, 95% confidence interval [0.027, 0.132]). In summary, market dynamics moderated the indirect effect of lean startup strategy on sustainable development via organizational iterative learning. Hypothesis 4a was thus supported.

**Table 6:** Bootstrapping results for moderated mediation effect.

Moderator	Level	Estimate	BootSE	LLCI	ULCI
Market dynamics	Low (-1SD)	0.066	0.031	0.027	0.132
	High (+1SD)	0.110	0.054	0.040	0.218
Technology dynamics	Low (-1SD)	0.118	0.037	0.059	0.206
	High (+1SD)	0.087	0.049	0.039	0.141

This research further examined whether technology dynamics moderated the mediating effect of organizational iterative learning on the relationship between lean startup strategy and sustainable development. As seen in Table 6, under conditions of low technology dynamics, lean startup strategy had a significant mediating effect on sustainable development via organizational iterative learning (estimate = 0.118, BootSE = 0.037, 95% confidence interval [0.059, 0.206]). Under conditions of high technology dynamics, the mediating effect of lean startup strategy on sustainable development via organizational iterative learning was significant but below low technology dynamics (estimate = 0.087, BootSE = 0.049, 95% confidence interval [0.039, 0.141]). Therefore, technology dynamics moderated the indirect effect of lean startup strategy on sustainable development via organizational iterative learning. Hypothesis 4b was thus supported.

### 4.5 Robustness test

Firstly, to further test the mediating effect, we utilized the bootstrap method with deviation correction by carrying out 5000 extraction repetitions and verifying the significance of the mediation path’s 95% confidence interval [62]. The results revealed that the mediating effect possessed a 95% confidence interval of [0.036, 0.179], excluding 0, indicating that the mediating effect was significant. Therefore, Hypothesis 2 was further supported.

Secondly, we employed a two-stage regression method to examine the potential endogeneity between organizational iterative learning and environmental dynamics. The dependent variable of organizational iterative learning was regressed with a moderating variable to obtain the residual item. Next, the residual item was taken as an alternative variable for organizational iterative learning, and the interaction item between the alternative variable and moderating variable was included in the regression equation. The results were then compared with the regression results for moderating effects in Table 5, and it was found that the significance results were consistent with those in the table. These findings suggest that the endogeneity problem could be ignored.

## 5. Discussion

### 5.1 Research conclusion

Based on 325 valid samples collected over two stages, this study found that lean startup strategy promoted sustainable development of new ventures via the path of enhanced organizational iterative learning. Market dynamics improved the positive effect of organizational iterative learning on sustainable development and technology dynamics weakened the positive effect of organizational iterative learning on sustainable development. Moreover, market dynamics/technology dynamics moderated the mediating effect of organizational iterative learning on the lean startup strategy—sustainable development relationship.

### 5.2 Theoretical contributions

First, this research linked lean startup strategy with sustainable development of new ventures, thus enriching both theoretical and empirical research on the contextual antecedents of sustainable development. Different from traditional entrepreneurship modes, lean startup strategy helps entrepreneurs quickly iterate products or services in a complex and diverse external environment to meet the dynamic needs of users [63, 64], this not only avoids wasting resources caused by blind development but also reduces entrepreneurial risks. The findings of this research affirm the importance of lean startup strategy for new ventures, with the increasing rigidity of entrepreneurial thinking and the existence of learning ability traps, lean startup strategy is gradually becoming an important prerequisite factor in the field of entrepreneurship. On the one hand, lean startup has broken through the industrial logic of "investigate first, then develop" [65], driving entrepreneurs to closely link product development with user needs, and subvert the previous R&D practices. On the other hand, lean startup solves the problem of resource constraints to a certain extent through user-driven orientation and resource patchwork, thereby avoiding the abortion of ideas in the later stage due to the large amount of resource investment in the early stage. But not all studies have found lean startup strategy to have favorable outcomes, because the implementation of the lean startup strategy increases employees’ innovation autonomy and self-management authority, it may be seen as a source of workplace stress. By illustrating the positive relationship between lean startup strategy and sustainable development, this study deepens our understanding of the concept of lean startup and provides empirical evidence that lean startup strategy has positive impacts on enterprise’s sustainability. Furthermore, while existing research on lean startup has focused on the manufacturing industry, this study focuses on the R&D department for technology new ventures distributed across different industries, thus expanding the scope of the survey subjects and enriching our understanding of lean startup strategies in various industries.

Second, this research explored the ways in which lean startup strategy influenced sustainable development from the perspective of organizational iterative learning. From the perspective of organizational learning, lean startup strategy predisposes entrepreneurs to maintain an iterative mindset, actively seek information feedback and carry out iterative learning. Although previous studies have emphasized the importance of iterative innovation [66], there is a lack of necessary explanation for the formation and cultivation of learning behaviors before innovation capacity building. Therefore, this study believes that lean startup strategy, as an important antecedent for triggering iterative learning in organizations, has higher requirements for the learning ability of entrepreneurs based on its confirmatory learning criteria [67], requiring them to strengthen their learning of user feedback, thereby triggering the processes of responsive learning, interactive learning, trial-and-error learning, and continuous learning, improving product development capabilities and promoting sustainable entrepreneurship in the cycle. Thus, this study reveals the mediating role and path mechanism of organizational iterative learning between lean startup and sustainable development, which enriches the theoretical research on organizational iterative learning.

Third, this research examined the moderating effect of market dynamics/technology dynamics on the relationship between organizational iterative learning on sustainable development and identified the boundary conditions of organizational learning with respect to enterprise resources. The results show that organizational iterative learning does not maintain the same effectiveness under any conditions, after incorporating environmental dynamics into the research model as a moderating variable, it is found that it increases with the increase of market dynamics and decreases with the increase of technology dynamics, this is somewhat different from the conclusion in previous studies that environmental dynamics can positively moderate performance. The generation of sustainability is not only influenced by environmental stimuli but also depends on whether an enterprise can integrate and utilize the available resources. This research tested the moderating effect of environmental dynamics, thus enriching our understanding of the boundary conditions of organizational iterative learning and providing a more fine-grained understanding of the relationship between organizational learning and new venture’s development. In terms of market transaction theory, existing studies have focused more on the direct impact of market stability on organizational coping, ignoring the fact that the choice of technical coping strategies is also related to organizational learning resources. By exploring the moderating role of environmental dynamics, this study emphasizes the significance of technology availability to organizational iteration.

### 5.3 Managerial implications

First, in the era of the new industrial revolution, new ventures need to establish effective sustainable development advantages. In the Notice on Building the Third Batch of Mass Entrepreneurship and Innovation Demonstration Bases, the General Office of the State Council of China proposed to focus on supporting innovative small and medium and micro enterprises to grow into important sources of innovation, and strive to build a gathering platform for lean startup. Evidently, lean practice has become an inevitable theme for contemporary new enterprises, and entrepreneurs need to attach importance to and focus on promoting lean startup strategies, adopt measures such as "employees and customers face to face" to cultivate employees’ demand-oriented thinking, encourage employees to exploit potential opportunities in the market by using rapid iteration and feedback traceability, and thereby improve the entrepreneurial learning capability and development performance of enterprises. Establishing business insight in the process of response, interaction, trial and error, and fully transform its own capability into sustainable product or service supply.

Second, organizational iterative learning represents enterprises’ capability to integrate and utilize the available resources and is a key factor in encouraging enterprises to establish a sustainable development order. On the one hand, managers should actively create a knowledge atmosphere that brings forth the new through the old, such as establishing the working mechanism for front-line employees to directly connect with customers, and broadening the access rights of employees to resources, etc., so that the organization can grasp market information in a more timely manner and ensure that the organization is always at the forefront of iterative learning. On the other hand, the results also suggest that managers should focus on demand-ability fit when designing jobs. When in high market dynamics, the organization should vigorously support iterative learning, empower employees to make independent decisions, and improve the organization’s innovation incubation rate and marketization degree, entrepreneurs should also pay attention to the contribution of employees and broaden the channels for employees to obtain resources, thus transforming the threat brought by environmental uncertainty into opportunities for the generation of creativity.

### 5.4 Limitations and future directions

With respect to methodology, first, all sample data in this research was derived from individual subjective evaluation, although we had weakened the impact of common method bias through a two-stage questionnaire collection procedure and emphasizing the confidentiality and anonymity of the survey, there were still individual biases that cannot be avoided. In future studies, the diary method can be used to continuously record observation results for 5 days to eliminate the accidental impact of individual bias. Second, the survey sample referenced by this study is aimed at technology new ventures, and it is uncertain whether the research conclusions can be extended to other types of enterprises, such as knowledge-based enterprises or non R&D enterprises. Future research should collect data from different types of enterprises on a broader range to verify these conclusions. Third, the number of survey samples involved in this study was limited. Due to the particularity of the research group, the results of this study cannot represent the situation of all new enterprises, so the research results need to be verified with a larger sample size.

With respect to theory, this study focused on the mediating role of organizational iterative learning in the relationship between lean startup strategy and sustainable development. When lean startup strategy, and organizational iterative learning were included in the regression model, lean startup strategy continued to positively predict sustainable development, indicating that other paths for the effect of lean startup strategy on organizational iterative learning may exist. In the future, we can explore other mediating variables such as opportunity recognition and failure learning to more comprehensively reveal the path mechanisms of the two. In addition, this study only discussed the contingency impact of environmental dynamics in the process of iterative learning, but the iterative learning process for enterprises is often affected by internal boundary characteristics such as organizational inertia, organizational resilience. Therefore, future research can be conducted considering these perspectives to further enrich the research model.

## Supporting information

S1 File Data. (XLS)
